# C19orf10 promotes malignant behaviors of human bladder carcinoma cells via regulating the PI3K/AKT and Wnt/β-catenin pathways

**DOI:** 10.7150/jca.56993

**Published:** 2021-05-19

**Authors:** Shi Li, Longyi Mao, Fangrong Zhao, Juan Yan, Guanbin Song, Qing Luo, Zesong Li

**Affiliations:** 1 College of Bioengineering, Chongqing University, Chongqing 400030, P. R. China.; 2 Guangdong Key Laboratory of Systems Biology and Synthetic Biology for Urogenital Tumors, Shenzhen Second People's Hospital, First Affiliated Hospital of Shenzhen University, Shenzhen, Guangdong 518000, P.R. China.; 3 Shenzhen Key Laboratory of Genitourinary Tumor, Shenzhen Second People's Hospital, First Affiliated Hospital of Shenzhen University, Shenzhen, Guangdong 518000, P.R. China.; 4 College of Chemical and Biological Engineering, Hunan University of Science and Engineering, Yongzhou, Hunan 425199, P.R. China.

**Keywords:** bladder cancer, C19orf10, proliferation, migration and invasion, PI3K/AKT, Wnt/β-catenin

## Abstract

**Background:** Chromosome 19 open reading frame 10 (C19orf10) is a myocardial repair mediator overexpressed in hepatocellular carcinoma. However, its function and clinical value in bladder cancer (BC) have not been reported. This study aimed to investigate the role of C19orf10 in BC progression and explore underlying mechanisms.

**Methods:** C19orf10 expression in BC tissues and human BC cell lines was assessed by reverse transcription-quantitative polymerase chain reaction (RT-qPCR) and western blot analysis. The correlation between the C19orf10 protein levels determined by immunohistochemical staining and the clinicopathological characteristics of 192 BC patients was evaluated. BC cell lines SW780, J82 and UMUC-3 were transfected with small interfering RNA (siRNA) targeting C19orf10 or plasmids overexpressing C19orf10. Cell proliferation, migration and invasion were measured by Cell Counting Kit-8, Colony formation, EdU incorporation and Transwell assays. The effect of small hairpin RNA (shRNA)-mediated stable C19orf10 knockdown on tumor formation was assessed in a xenograft mouse model. The expressions of epithelial-mesenchymal transition (EMT) markers, PI3K/AKT and Wnt/β-catenin signaling pathways-related molecules were determined by western blot assay.

**Results:** C19orf10 was significantly upregulated in the BC tissues and a panel of human BC cell lines. High expression of C19orf10 was positively associated with malignant behaviors in BC. C19orf10 knockdown inhibited cell proliferation, migration, and invasion in SW780 and J82 cells, while C19orf10 overexpression in UMUC-3 cells resulted in opposite effects. In addition, C19orf10 silence in SW780 cells suppressed tumor growth in xenograft mice. Moreover, C19orf10 promotes the malignant behaviors and EMT of human bladder carcinoma cells via regulating the PI3K/AKT and Wnt/β-catenin pathways.

**Conclusion:** C19orf10 is overexpressed in BC and functions as an oncogenic driver that promotes cell proliferation and metastasis, and induces EMT of BC cells via mechanisms involving activation of the PI3K/AKT and Wnt/β-catenin pathways. This study provides valuable insight on targeting C19orf10 for BC treatment.

## Introduction

Bladder cancer (BC) is one of the most prevalent malignancies of the urinary system, and its incidence and mortality rates both rank first among male genitourinary tumors in China 1, 2. BC can be divided into muscle-invasive bladder carcinoma (MIBC) and nonmuscle-invasive bladder carcinoma (NMIBC), depending on the degree of invasion; the depth of invasion largely affects the prognosis and management of the disease. Although approximately 80% of cases are diagnosed early as NMIBC, up to 70% of patients with NMIBC have at least one recurrence after surgical treatment 3. The degree of malignancy for MIBC is higher, and the 5-year survival rate of patients with metastatic or locally advanced disease is only about 15% 4. Despite the treatment strategies of BC, including robotic surgical techniques and immunotherapy, have been advanced significantly in recent years, the overall survival rates of patients with BC have not improved much 5, 6. Therefore, new molecular biomarkers and therapeutic targets for BC treatment are urgently needed.

Chromosome 19 open reading frame 10 (C19orf10), a novel protein also known as myeloid-derived growth factor (MYDGF), is a paracrine-acting protein derived from monocytes and macrophages 7. C19orf10 is highly conserved in evolution and is mainly localized in the Golgi and endoplasmic reticulum, but it functions extracellularly 8. The N-terminus and C-terminus of human C19orf10 contain a signal peptide and a conserved sequence of the endoplasmic reticulum, respectively, but human C19orf10 has no sequence homology with any other proteins 9. Structural analysis has revealed that C19orf10 consists of 10 antiparallel β-strands forming a β-sandwich, and its folding topology is not similar to other cytokines or growth factors 10. Furthermore, functional studies have demonstrated that C19orf10 promotes angiogenesis 11, 12, inhibits myocardial cell apoptosis, promotes vascular endothelial cell proliferation, and plays a role in cardiac repair 7, 13. Although it has been speculated that C19orf10 has an effect on synovial biology 14 and may also be involved in the regulation of adipose tissue development 15, more evidence is needed to further confirm these notions.

So far, there are few reports on the role of C19orf10 in the initiation and development of tumors. It has been shown that C19orf10 overexpression can promote the proliferation of hepatocellular carcinoma cells and activate the AKT signaling pathway 16. In addition, it has been reported that C19orf10 contributes to the proliferation of lymphocytes 17. However, the relationship between C19orf10 expression and tumor progression remains largely elusive in many types of cancer. In our previous study, we showed that C19orf10 was expressed at higher levels in BC tissues in comparison to matched adjacent normal tissues by transcriptome analyses 18. Nevertheless, whether the expression level of C19orf10 is associated with the progression of BC remains unknown.

In the present study, we further analyzed the expression levels of C19orf10 in normal bladder epithelial cells and BC tissues from different cohorts of patients as well as in some common human BC cell lines and examined the relationship between C19orf10 expression and the clinicopathological characteristics of BC patients. Moreover, through small interfering RNA (siRNA) transfection-mediated silencing of C19orf10 and plasmid transfection-mediated overexpression of C19orf10, we studied the functions of C19orf10 in BC cell proliferation, migration, and invasion. The *in vivo* effect of stable C19orf10 knockdown on tumor formation was also assessed in a xenograft mouse model with nude mice. Furthermore, we explored the preliminary molecular mechanisms underlying the oncogenic role of C19of10 in BC cells.

## Materials and methods

### Clinical samples and ethics statement

Forty-two pairs of cancerous and matched normal (at least 3 cm away) bladder epithelial tissues were collected from BC patients who underwent a radical cystectomy at The First Affiliated Hospital of Shenzhen University (Shenzhen, China). All patients were pathologically diagnosed as having BC, and no patients received radiotherapy, chemotherapy, or immunotherapy before surgical treatment. Samples were snap-frozen in liquid nitrogen and stored at -80 °C. All patients signed informed consent forms prior to the use of their clinical materials, and this study was approved by the Ethics Committee of The First Affiliated Hospital of Shenzhen University.

### Cell culture

The following cell lines were purchased from the American Type Culture Collection (ATCC, Manassas, VA, USA): human BC cell lines SW780 (ATCC® CRL-2169), UMUC3 (ATCC® CRL-1749), 5637 (ATCC® HTB-9), T24 (ATCC® HTB-4), TCCSUP (ATCC® HTB-5™), and J82 (ATCC® HTB-1), and the immortalized urothelial cell line SV-HUC-1 (ATCC® CRL-9520). All cell lines were authenticated by short tandem repeat DNA profiling analysis and tested as being free from mycoplasma contamination by the vender. SW780 and 5637 cells were cultured in Roswell Park Memorial Institute (RPMI)-1640 medium; T24 cells were cultured in McCoy's 5A medium; J82, UMUC3, and TCCSUP cells were cultured in Minimum Essential Medium (MEM); and SV-HUC-1 cells were cultured in F-12K medium. All media (Gibco, Waltham, MA, USA) were supplemented with 10% fetal bovine serum (Gibco) and 1% penicillin/streptomycin (Gibco). Cells were cultured at 37 °C in a humidified atmosphere of 5% CO_2_.

### RNA isolation and reverse transcription-quantitative polymerase chain reaction (RT-qPCR)

Total RNA was extracted from the cell samples or BC tissue samples using Trizol reagent (Thermo Fisher Scientific, MA, USA), according to the manufacturer's protocol. Complementary DNA (cDNA) synthesis was performed using a ReverTra Ace™ qPCR RT Kit (TOYOBO, Osaka, Japan), according to the manufacturer's instructions. The mRNA levels of target genes in the tissue samples and cell lines were analyzed by relative fluorescence quantification using TB Green™ Premix Ex Taq™ II (Tli RNaseH Plus) Master Mix (Cat. # RR820A; TaKaRa, Shiga, Japan). The relative gene expression of C19orf10 was normalized to the level of β-actin using the comparative threshold cycle (2^-ΔΔCT^) method. All qPCR reactions were conducted in triplicate. The primer sequences of C19orf10 and β-actin are shown in Supplementary Table S1.

### Western blot analysis

Cells and BC tissues were washed with ice-cold phosphate-buffered saline and lysed in precooled radioimmunoprecipitation assay (RIPA) lysis buffer (Thermo Fisher Scientific) plus 1% protease inhibitors (Sigma-Aldrich, St. Louis, MO, USA). The total protein concentration was determined by a Pierce BCA Protein Assay Kit (Pierce Biotechnology, Rockford, IL, USA). Samples containing equal amounts of protein (20 μg for each lane) were separated by electrophoresis on 12% sodium dodecyl sulfate (SDS)-polyacrylamide gels and transferred to polyvinylidene difluoride (PVDF) membranes (Millipore, Billerica, MA, USA). The nonspecific protein interactions were blocked by incubating the membranes with 5% nonfat milk at room temperature for 2 h. The membranes were subsequently incubated with primary antibodies (the antibody information is shown in Supplementary Table S2) overnight at 4 °C. After washing the membranes with 1× Tris-buffered saline supplemented with 0.1% Tween® 20 detergent, they were further incubated with a horseradish peroxidase (HRP)-conjugated secondary antibody at room temperature for 1 h. The proteins of interest were detected using an enhanced chemiluminescence (ECL) kit (EMD Millipore, Burlington, MA, USA) and visualized with the GeneGnome XRQ Chemical Imaging System (Gene Company Limited, Hong Kong, China). β-actin or β-tubulin was used as an indicator of total protein loading for all the western blots.

### Immunohistochemical staining

A human tissue microarray containing 192 pairs of paraffin-embedded tissue samples was purchased from Alenabio Technology Co., Ltd. (Xi'an, China). Immunohistochemical staining was performed on the tissue microarray to assess the protein levels of C19orf10. After dewaxing, the tissue microarray was treated with sodium citrate solution (10 mM, pH 6.0) for antigen repair in a microwave oven (high temperature for 2 min, medium temperature for 5 min, and low temperature for 10 min). Then the tissue samples were blocked with 3% hydrogen dioxide solution and stained with anti-C19orf10 antibody (1:400 dilution, Proteintech Group Inc., Wuhan, China), anti-PCNA (1:200 dilution, Santa Cruz Biotechnology, Inc., Santa Cruz, CA, USA), and anti-Ki67 antibody (1:400 dilution, Abcam, Cambridge, MA, USA) followed by incubation with biotin-conjugated goat anti-rabbit and biotin-conjugated goat anti-mouse secondary antibody (Fuzhou Maixin Biotech Co., Ltd., Fuzhou, China) and staining with 3,3ʹ-diaminobenzidine (Fuzhou Maixin Biotech Co., Ltd.). Finally, the nuclei were re-stained with hematoxylin, and images were obtained with an upright microscope system (CX41; Olympus, Tokyo, Japan).

### Transcriptome sequencing and database analysis

Differentially expressed genes between the BC tissues and the matched adjacent normal tissues (n = 9 pairs) were identified by transcriptome sequencing, as reported previously 18. The RNA expression data of C19orf10 was profiled based on normalized RNA-seq expression data sets of BLCA in TCGA database (TCGA_BLCA_exp_HiSeqV2-2015-02-24) from the UCSC Xena Browser (https://xenabrowser.net/datapages/). It was used to determine the mRNA expression changes of related genes in the BC tissues and the matched adjacent normal tissues.

### siRNAs and plasmids transfection

The oligonucleotides of three siRNAs and negative control siRNA were purchased from RIBOBIO Inc (Guangzhou, China), and the sequences are listed in Supplementary Table S3. The SF20 (MYDGF) (NM_019107) Human Tagged ORF Clone plasmid vector and negative control plasmid vector were purchased from OriGene Technologies Inc (Rockville, MD, USA). These siRNA oligonucleotides and plasmids were transfected at using Lipofectamine 3000 (Thermo Fisher Scientific). The total RNA and proteins were extracted at 24-72 h after transfection.

### Lentiviral (LV) transfection of cells

C19orf10 shRNA interference lentiviral vector was constructed and synthesized by Genepharma (Suzhou, China). The lentivirus was transfected into cells according to the manufacturer's instructions. Cells were seeded (1×10^5^ cells/ml) into 6-well plates and incubated for 24 h to reach 50% confluence, and then replaced with fresh medium containing lentiviral supernatant (MOI=20) and 5 μg polybrene. Successfully infected sh-C19orf10 cells were green fluorescent positive when observed under fluorescence microscope after 72 h, and these positive cells were cultured in puromycin-containing medium (2 μg/ml) for 7 days. The interference efficiency of C19orf10 was determined using western blot assay.

### Cell counting kit-8 (CCK-8) assay

At 48 h after transfection of the siRNA oligos, the cells were seeded into 96-well plates at a density of 2000 cells per well. After incubation for 0, 24, 48, and 72 h, respectively, the cell viability was assessed by the cell counting kit-8 (CCK-8; Dojindo, Kumamoto, Japan), according to the manufacturer's instructions. Briefly, the CCK-8 reagent was added to the culture medium, the cells were incubated for 2 h at 37 °C, and the absorbance was measured at 450 nm.

### Colony formation assays

The colony formation assay was performed by seeding cells into 6-well plates (1000 cells/well) and incubating the cells under routine conditions for 1-2 weeks. The plates were washed twice with phosphate-buffered saline and fixed with 4% paraformaldehyde for 15 min at room temperature. After staining with 0.1% crystal violet for 20 min, the cells were photographed and the numbers of colonies were counted.

### 5-ethynyl-2'-deoxyuridine (EdU) incorporation assay

Briefly, cells were seeded in 96-well plates, then incubated with 50 μM EdU for 2 hours and stained with Apollo® fluorescent dye, according to manufacturer's instructions (Cell-Light EdU Apollo567 *In vitro* Kit, Ribobio, China). Images were acquired under a fluorescent microscope at 567 nm excitation.

### Cell migration and invasion assays

A 200 µL volume of serum-free medium containing approximately 4 × 10^4^ cells was seeded into the upper chamber of a transwell (8-μm pores, BD Biosciences, Franklin Lakes, NJ, USA), and the lower chamber was filled with 500 µL of culture medium containing 10% fetal bovine serum as a chemoattractant. The invasion assay method was similar to that of the migration assay, except that the chambers had a porous membrane coated with Matrigel (BD Biosciences). In brief, after incubation at 37 °C for 24 h, the BC cells remaining on top of the transwell were scraped off with a cotton swab, and the successfully translocated cells were fixed with 4% paraformaldehyde for 15 min. Subsequently, the cells were stained with 0.1% crystal violet for 20 min, and the amount of cell migration and invasion was analyzed in five random fields under a light microscope.

### Subcutaneous tumor xenografts in nude mice

Male BALB/c nude mice (4-5 weeks, 16-18 g) were purchased from Guangdong Medical Animal Experimental Center. Fifteen mice were divided into three groups with 5 mice in each group. 5 × 10^6^ cells were inoculated subcutaneously on the right side of each group. Once the tumor xenografts emerged, mice body weight and tumor size were measured once every other day. Mice were sacrificed before the transplanted tumors were excised. The tumor volume was calculated by a standard formula: V= 1/2 (length × width^2^). All animal experiments involving the use of mice in this study have been approved by the Experimental Animal Welfare Ethics Committee of the Shenzhen Peking University Hong Kong University of Science and Technology Medical Center.

### Statistical analysis

The data were expressed as the mean ± standard deviation. All analyses were performed using GraphPad Prism, version 6 (GraphPad Software, San Diego, CA, USA). The paired *t* test was used to compare the differences between the BC tissues and the adjacent normal tissues. Correlations between the expression levels of C19orf10 and the clinicopathological features were assessed using the chi-squared test. The differences between variables in other experiments were analyzed by the independent Student's *t* test. A *P* value less than 0.05 indicated statistical significance.

## Results

### C19orf10 expression is upregulated in human BC tissues and cells

**Figure 1:**
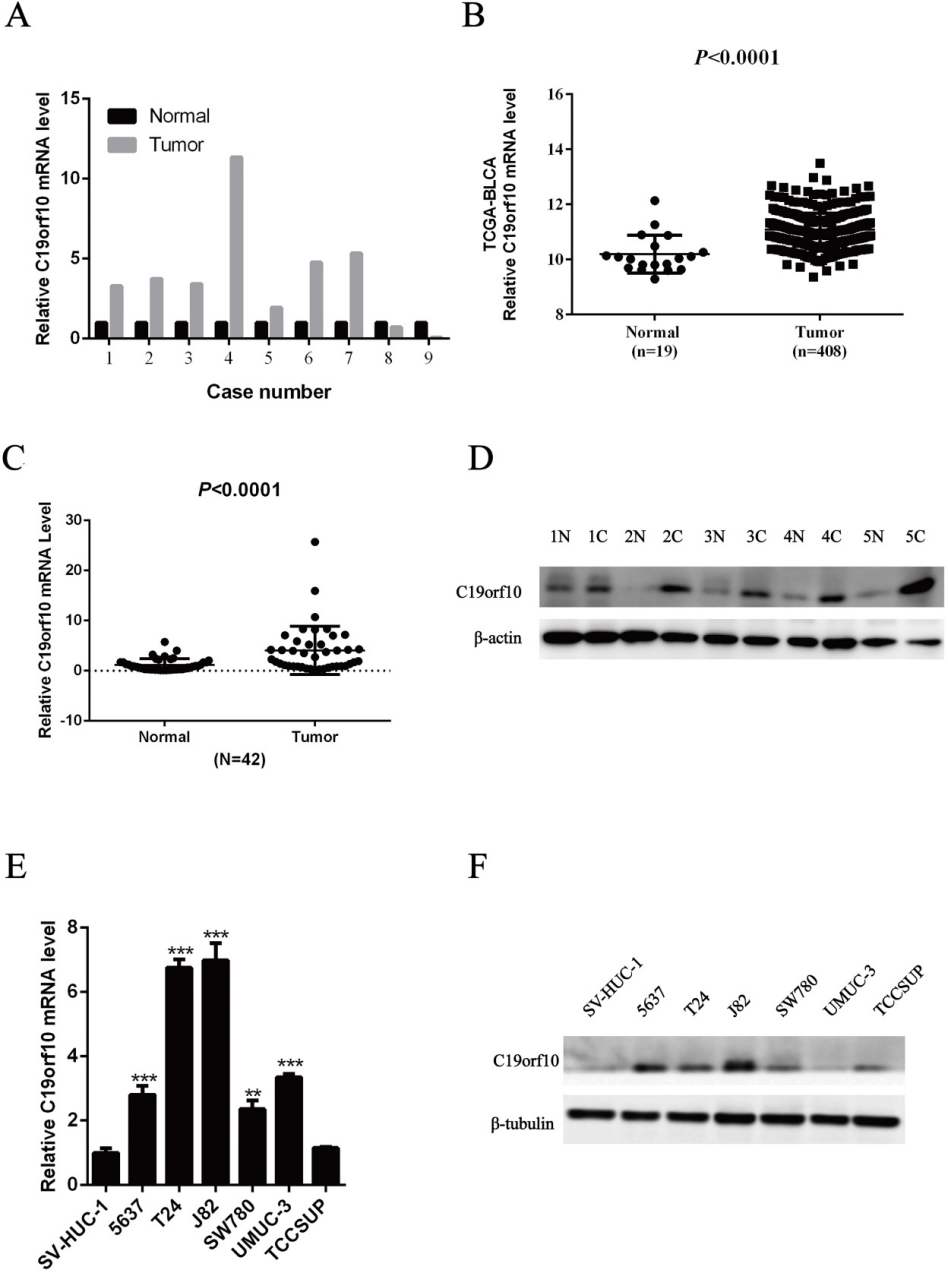
** High expression of C19orf10 in bladder carcinoma tissues and bladder carcinoma cell lines.** (A) Transcriptome sequencing was performed to analyze the C19orf10 mRNA levels in nine pairs of BC tissues and adjacent normal tissues. The expression level of C19orf10 in the adjacent normal tissue of each pair was set as 1, and the relative expression level in the BC tissue was calculated. (B) The expression levels of C19orf10 in BC tissues and normal bladder tissues from The Cancer Genome Atlas database were compared. (C) The C19orf10 mRNA levels in tissues from 42 pairs of BC and adjacent tissues were analyzed by qPCR. (D) The C19orf10 protein levels in five pairs of BC tissues and adjacent normal tissues were determined by western blot. (E) The C19orf10 mRNA levels in the normal bladder epithelial cell line SV-HUC-1 and six different BC cell lines were analyzed using qPCR. n = 3 for each group; **P < 0.01, ***P < 0.001, compared with the SV-HUC-1 group. (F) The C19orf10 protein levels in SV-HUC-1 cells and six different BC cell lines were determined by western blot analysis. Representative western blot images are shown.

First, we determined the mRNA expression levels of C19orf10 in human BC tissues by examining our previous transcriptome sequencing results. It was found that the C19orf10 mRNA level in BC tissues was significantly higher than that in the adjacent normal tissues (Fig. 1A). The RNA expression data of C19orf10 was profiled based on normalized RNA-seq expression datasets of BLCA in TCGA database (TCGA_BLCA_exp_HiSeqV2-2015-02-24) from the UCSC Xena Browser (https://xenabrowser.net/datapages/). It also demonstrated that the human BC samples had markedly more expression of C19orf10 than the normal tissues (Fig. 1B), which is consistent with our sequencing results. To verify these findings, we measured the expression levels of C19orf10 in 42 pairs of cancer tissues and noncancerous surrounding tissue from patients with BC. The qPCR results indicated that the mRNA level of C19orf10 in the BC tissues was significantly higher than that in the adjacent normal tissues (Fig. 1C). In addition, the protein expression of C19orf10 in the BC tissues was also much more than that in the normal tissues, as revealed by western blot assays (Fig. 1D; n = 5). Taken together, C19orf10 is overexpressed in human BC tissues in comparison to the adjacent normal tissues in patients. To determine the expression of C19orf10 in human BC cell lines, we evaluated the mRNA and protein expression levels of C19orf10 in normal urothelial cells (SV-HUC-1) and six BC cell lines (SW780, UMUC3, 5637, T24, TCCSUP, and J82). Our qPCR (Fig. 1E) and western blot (Fig. 1F) analyses revealed that compared with the normal urothelial cells, the BC cells had a significantly increased expression of C19orf10.

### C19orf10 expression is associated with the pathological grade of BC

**Figure 2:**
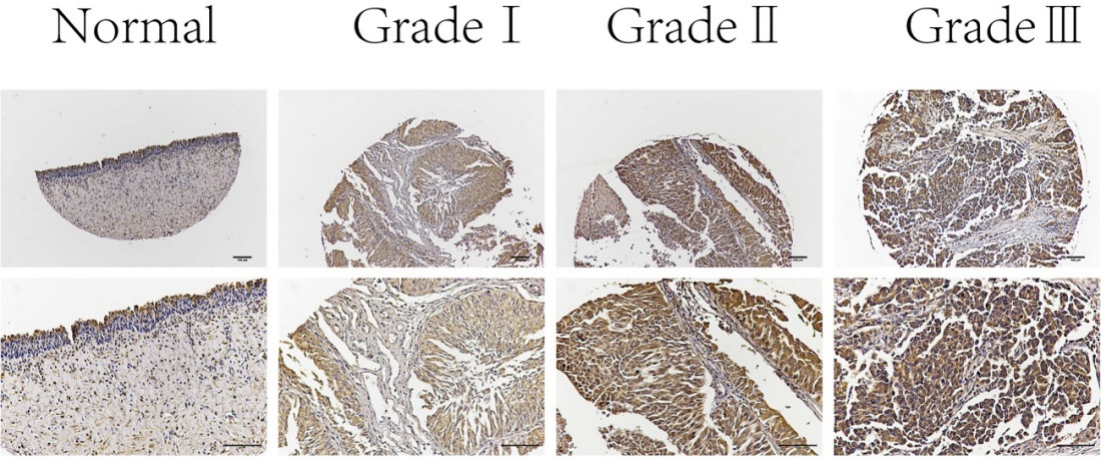
** Representative immunohistochemical images of C19orf10 expression in normal human bladder epithelial tissue and BC tissue from patients with different pathological grades.** Scale bar, 100 µm.

Next, we examined C19orf10 expression in normal bladder epithelial tissues and BC tissues of different pathological grades by immunohistochemical staining. The results showed that C19orf10 expression in the BC tissues was higher than that in the normal bladder epithelial tissues and that the BC tissues with higher disease grades appeared to have more C19orf10 expression (Fig. 2). To further determine the clinical significance of C19orf10 overexpression, we analyzed the relationship between the clinical features and the C19orf10 protein levels in 192 BC patients (Table 1). We found that the expression level of C19orf10 was related to the tumor histological grade and that the histological grade was higher in the group with a greater expression of C19orf10. However, no statistically significant differences were found between the expression level of C19orf10 and other parameters, including the clinical tumor/node/metastasis classification, age, gender, and lymph node metastasis.

### C19orf10 knockdown suppresses migration and invasion of BC cells

**Figure 3:**
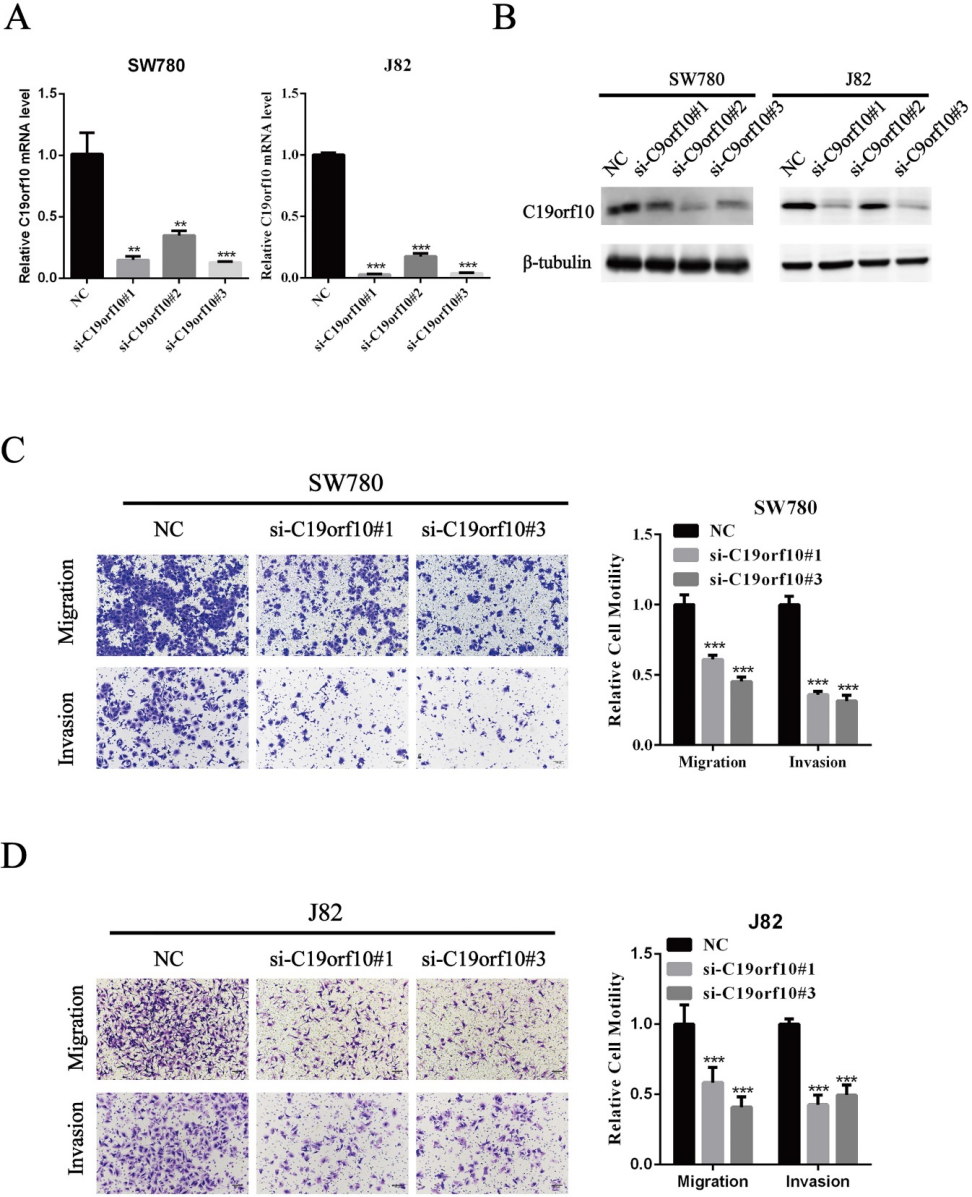
** Silencing of C19orf10 inhibited the migration and invasion of BC cells.** (A-B) The mRNA (A) and protein (B) expression levels of C19orf10 were determined by qPCR and western blot assays, respectively. **P < 0.01, ***P < 0.001. (C-D) Transwell assays were used to assess the migration and invasion of SW780 cells (C) and J82 (D) cells at 48 h after transfection of negative control or C19orf10-specific siRNA oligos. Representative images of cell migration and invasion in the transwell experiments are shown (C-D), and the relative cell motility was quantitated. n = 3 for each group; ***P < 0.001, compared with the control group.

Since C19orf10 was highly expressed in SW780 and J82 cancer cells, we set out to knock down C19orf10 expression and examined the effects of C19orf10 silencing on cell proliferation. All three C19orf10-specific siRNA oligos remarkably reduced the mRNA (Fig. 3A) and protein (Fig. 3B) expression of C19orf10 in both SW780 and J82 cells. The interfering efficiencies of si-C19orf10#1 and si-C19orf10#3 were better than that of si-C19orf10#2 in both cell lines, so they were used in the following experiments. The motility and invasion abilities of cells are crucial for the progression and metastasis of cancer. Therefore, transwell chamber assays were used to investigate whether C19orf10 knockdown can affect the migration and invasion of BC cells. We found that both SW780 (Fig. 3C) and J82 (Fig. 3D) cells displayed dramatically reduced migration and invasion abilities after silencing C19orf10 by transfection of siRNA oligos.

### C19orf10 knockdown inhibits the proliferation and colony formation of BC cells

**Figure 4:**
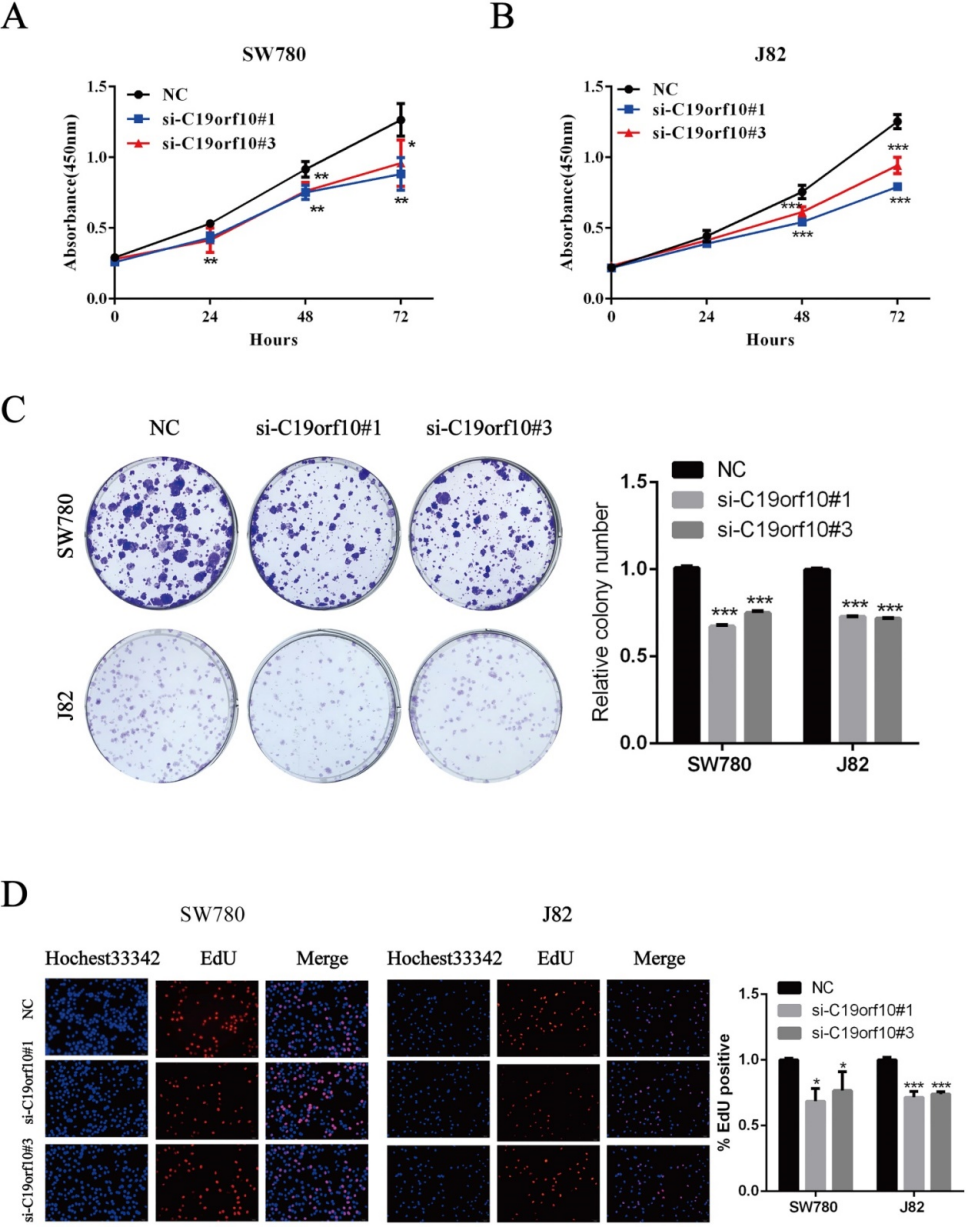
** Silencing of C19orf10 inhibited the proliferation and colony formation of BC cells.** (A-D) SW780 and J82 cells were transfected with negative control or C19orf10-specific siRNA oligos, and cell proliferation and colony formation were assessed at 48 h after transfection. (A-B) The cell viability after silencing C19orf10 in SW780 (A) and J82 (B) cells was measured using the CCK-8 assay. *P < 0.05, **P < 0.01, ***P < 0.001. (C)The cell proliferation rates of SW780 cells and J82 cells after silencing C19orf10 were assessed using a colony formation assay, and the number of cell colonies was normalized to that of the negative control group. n = 3 for each group; ***P < 0.001, compared with the control group. (D) Detection of cell proliferation by EdU incorporation assay. The relative proliferation rate was quantitated by calculating the percent of EdU-positive cells. *P < 0.05, ***P < 0.001.

The proliferation of BC cells after transfection of control or C19orf10-specific siRNA oligos was first determined by the CCK-8 method, which showed that C19orf10 knockdown led to a significantly decreased cell proliferation in a growth-time dependent manner in both SW780 (Fig. 4A) and J82 (Fig. 4B). Consistent with the CCK-8 results, the colony formation abilities of SW780 and J82 cells were also significantly suppressed after knockdown of C19orf10, compared with those of the negative control cells (Fig. 4C). In order to understand the effect of C19orf10 on the proliferation more intuitively, EdU incorporation experiment was used to DNA synthesis detection. Under the fluorescence field, the cell number of red fluorescence decreased in si-C19orf10#1 and si-C19orf10#3 groups, indicating that cells in the proliferative phase decreased and the cellular proliferation capacity was suppressed when C19orf10 expression was reduced (Fig. 4D). Therefore, C19orf10 silencing can markedly suppress the proliferation and colony formation of human BC cells.

### Overexpression of C19orf10 promotes the growth, migration, and invasion of BC cells *in vitro*

**Figure 5:**
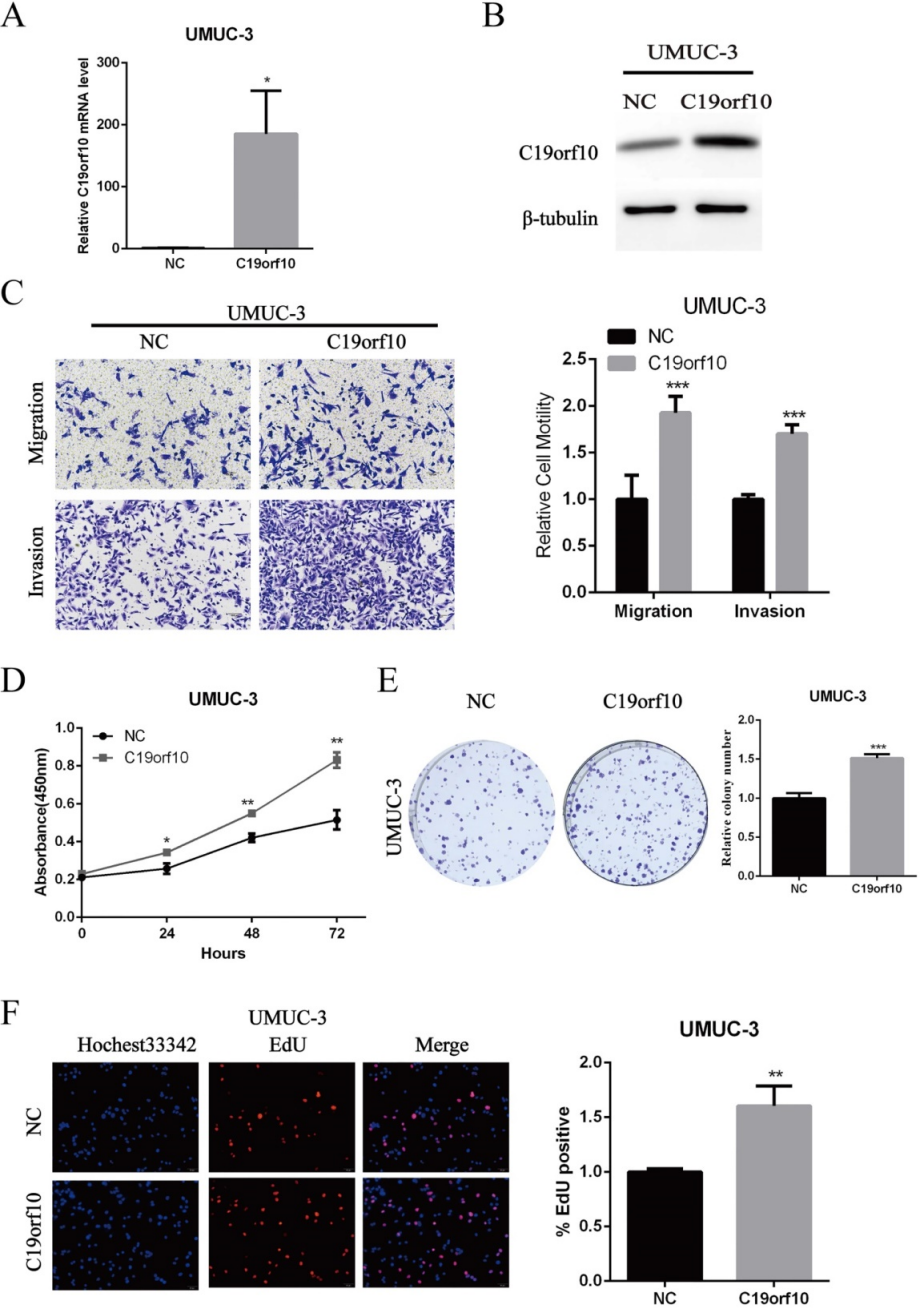
**Overexpression of C19orf10 promoted the proliferation, migration and invasion of BC cells**. (A-B) The mRNA (A) and protein (B) expression levels of C19orf10 in UMUC-3 cells after transfection of negative control (NC) or C19orf10-expressing plasmids were determined by qPCR and western blot assays, respectively. ***P < 0.001. (C) Transwell assays were used to assess the migration and invasion of UMUC-3 cells at 48 h after transfection of NC or C19orf10-expressing plasmids. Representative images of cell migration and invasion in the transwell experiments are shown (C), and the relative cell motility was quantitated. n = 3 for each group; ***P < 0.001, compared with the control group. (D) CCK-8 assay was performed to evaluate the cell viability for different days (1-3 day). *P<0.05, **P<0.01. (E) Colony formation assay was used to assess cell proliferation (left panel) and quantification (right panel) of formation by counting total colony numbers. ***P < 0.001. (F) Detection of cell proliferation by EdU incorporation assay. The relative proliferation rate was quantitated by calculating the percent of EdU-positive cells. **P<0.01.

Since C19orf10 was moderately expressed in UMUC-3 cells, we set out to overexpress C19orf10 and examine the effects of C19orf10 overexpression on cell malignant behaviors. Transfection of C19orf10-expressing plasmid into UMUC-3 cells remarkably increased the mRNA (Fig. 5A) and protein (Fig. 5B) levels of C19orf10 in UMUC-3 cells. Transwell chamber assays were used to investigate whether C19orf10 overexpression can affect the migration and invasion of BC cells. We found that UMUC-3 cells displayed dramatically increased migration and invasion abilities after overexpressing C19orf10 (Fig. 5C). CCK-8 assay also showed that C19orf10 overexpression led to a significantly increased cell proliferation in a growth-time dependent manner in UMUC-3 cells (Fig. 5D) cells. Consistent with the CCK-8 assay results, the colony formation ability of UMUC-3 cells was also significantly enhanced after overexpression of C19orf10, compared with that of the negative control cells (Fig. 5E). Moreover, the EdU assay also indicated that overexpression of C19orf10 induced cell proliferation (Fig. 5F). Therefore, C19orf10 overexpression can markedly promote the growth, migration, and invasion of human BC cells.

### Deficiency of C19orf10 inhibits the malignant growth of subcutaneous cancer xenografts *in vivo*

**Figure 6:**
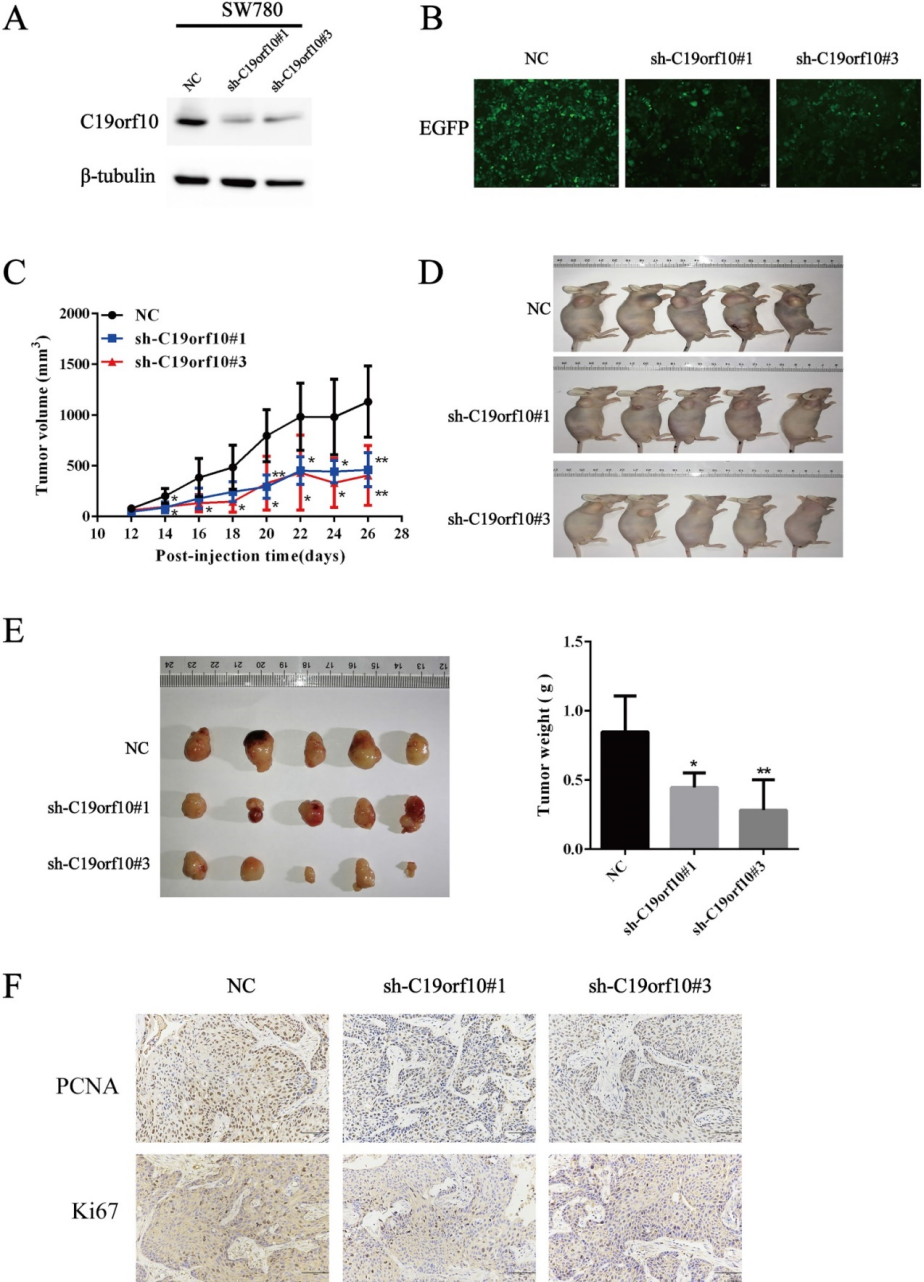
** Deficiency of C19orf10 inhibited the malignant growth of subcutaneous cancer xenografts *in vivo*.** (A) The protein levels of C19orf10 in stable SW780 cells with infection of control or C19orf10-shRNA-expressing lentivirus were determined by western blot assays. (B) The infection efficiency of lentivirus in the above cells was observed under a fluorescence microscope. (C) Growth curves show the tumor sizes in nude mice inoculated with the indicated SW780 cell lines (NC, sh-C19orf10#1, and sh-C19orf10#3 cells) during an observation time of four weeks. n=5 per group; *P < 0.05, **P < 0.01. (D-E) Mice were sacrificed at 4 weeks after tumor cells inoculation. The images of mice at end-point are shown (D), and the excised tumor tissues were images and weighed (E). *P < 0.05, **P < 0.01. (F) Representative images of immunohistochemical staining (IHC) analysis of PCNA and Ki67 protein levels in tumor tissues. Scale bars, 100 µm.

Knockdown of C19orf10 was proved to inhibit the malignant behaviors of tumor cells *in vitro*. Since anchorage-independent growth is a hallmark of tumorigenesis 19, an animal xenograft model was next used to test the effect of C19orf10 deficiency on the carcinogenesis of bladder cancer cells. First, we confirmed that SW780 cells were able to grow *in vivo* after subcutaneous inoculation into nude mice in our preliminary experiments. Western blot (Fig. 6A) and cell fluorescence (Fig. 6B) analysis confirmed that stable SW780 cell lines with infection of lentivirus expressing C19orf10-specific sh-RNA displayed reduced expression of C19orf10 protein in comparison to the control cells. Compared with mice inoculated with the control SW780 cells, mice inoculated with C19orf10 knockdown cells demonstrated significantly slower growth of xenografted tumors (Fig. 6C), and evidently smaller tumor sizes in the C19orf10 knockdown groups were identified at 4 weeks after inoculation (Fig. 6D). Compared with the control group, the C19orf10 knockdown groups had significantly reduced tumor weight (Fig. 6E). In addition, Ki67 and PCNA, proteins strictly associated with cell proliferation, were found at lower levels in tumor tissues from the sh-C19orf10 groups, compared to that in the control group (Fig. 6F). These results suggest that loss of C19orf10 inhibits the malignant growth of subcutaneous bladder carcinoma xenograft in nude mice.

### C19orf10 promotes the malignant behaviors and EMT of human bladder carcinoma cells via regulating the PI3K/AKT and Wnt/β-catenin pathways

**Figure 7:**
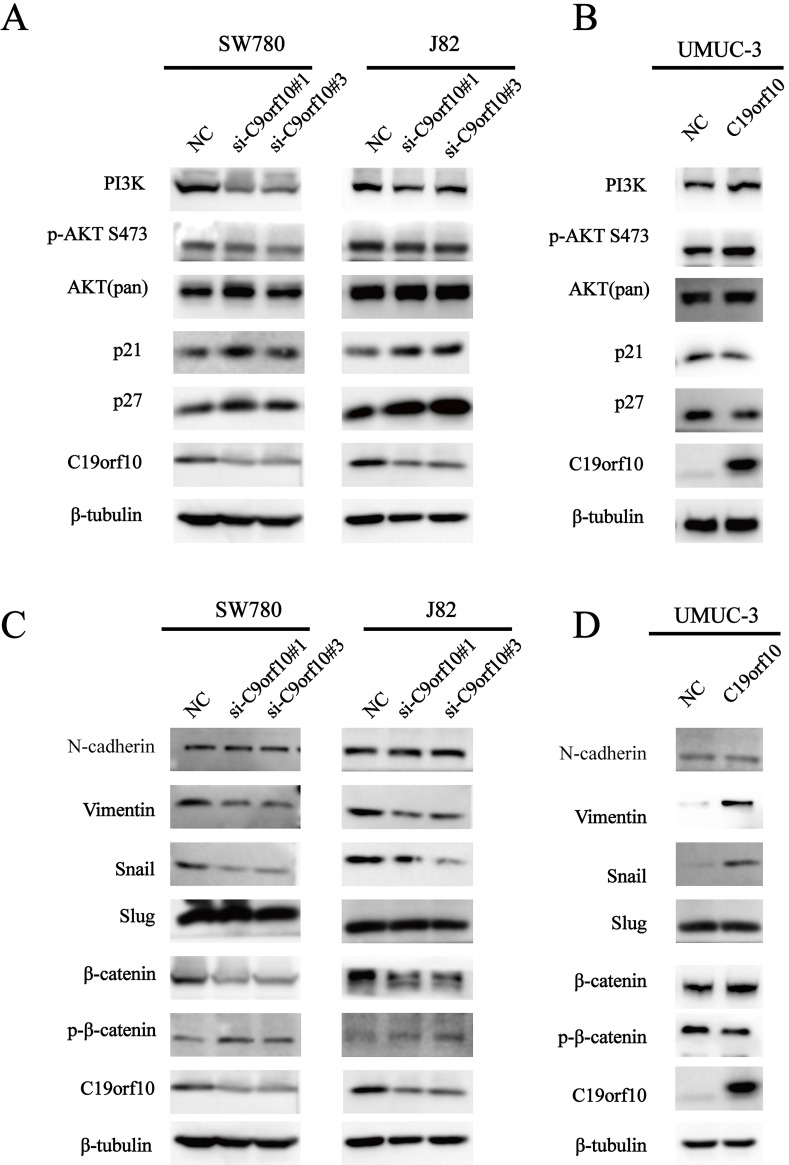
** C19orf10 promotes malignant behaviors and EMT of human bladder carcinoma cells via regulating the PI3K/AKT and Wnt/β-catenin pathway.** (A) Western blotting analysis of PI3K, p-AKT, total AKT, p21, and p27 expressions in control or C19orf10 siRNA-expressing SW780 and J82 cells. (B) Western blotting analysis of PI3K, p-AKT, AKT (pan), p21, and p27 expressions in control or C19orf10-overexpressed UMUC-3 cells. (C) Western blotting analysis of N-cadherin, Vimentin, Snail, Slug, β-catenin, and p-β-catenin expressions in control or C19orf10 siRNA-expressing SW780 and J82 cells. (D) Western blotting analysis of N-cadherin, Vimentin, Snail, Slug, β-catenin, and p-β-catenin in control or C19orf10-overexpressed UMUC-3 cells.

Our results indicate that loss of C19orf10 leads to significantly change in cell malignant behaviors, such as *in vitro* proliferation, migration, invasion, and *in vivo* overgrowth of the carcinoma xenografts derived from SW780 cells. Activation of AKT (protein kinase B) signaling can contribute to cell proliferation and tumor progression by modulating its downstream cell cycle factors 20. Furthermore, the promotion of cell proliferation by C19orf10 overexpression has been shown to be closely related to the AKT pathway in liver cancer 16. Hence, we measured the protein levels of important proteins involved in the phosphatidylinositol 3‑kinase (PI3K)/AKT signaling pathway. We found that the protein expression of PI3K, and phosphorylated AKT (p-AKT) was greatly downregulated, while the expression of p21 and p27 was drastically increased in SW780 and J82 cells transfected with C19orf10-specific siRNA oligos (Fig. 7A). In addition, C19orf10 overexpression in UMUC-3 cells resulted in the opposite changes on the protein levels of these molecules (Fig. 7B). Therefore, our preliminary exploration of the molecular mechanisms suggested that the PI3K/AKT signaling pathway is involved in the role of C19orf10 in conferring the malignant traits of BC cells.

Epithelial-mesenchymal transition (EMT) serves a critical role in tumor progression by transforming epithelial cells into a mesenchymal state 21. To determine whether C19orf10 inhibits BC invasion via regulating EMT, we tested specific biomarkers in the EMT process. Western blot results showed that knockdown of C19orf10 down-regulated the expression of Snail and Vimentin, while N-Cadherin expression was not affected (Fig. 7C), whereas C19orf10 overexpression caused the opposite changes (Fig. 7D). However, the protein expression of E-Cadherin was too low to be detected. There is evidence showing that cell migration and invasion are closely related to Wnt/β-catenin 22 and EMT signaling pathways 23, 24. Whether C19orf10 promotes the progression of BC through the Wnt/β-catenin signaling pathway is still unclear. Therefore, we determined the expression levels of β-catenin and p-β-catenin in BC cells with manipulated expression of C19orf10. While the silencing of C19orf10 significantly reduced the level of β-catenin and increased the protein level of p-β-catenin in SW780 and J82 cells (Fig. 7C), C19orf10 overexpression led to the opposite changes. In summary, these data indicate that the promotion of EMT by C19orf10 in bladder cancer may be related to its activation of Wnt /β-catenin signaling pathway.

## Discussion

Despite improved early diagnosis, resection, and targeted treatment techniques, there has been no significant improvement in the treatment of localized or metastatic BC over the past few decades, mainly because of the malignant properties of cancer cells such as unrestricted proliferation, metastasis, and resistance to apoptosis 24,25. Consequently, it was urgently desired to identify new molecular targets for BC.

C19orf10 is widely expressed in a variety of tissues, and current research regarding C19orf10 mainly focuses on its role in the cardiovascular system. C19orf10 could improves tissue repair and heart function after myocardial infarction 7,13. However, few articles have involved the functions of C19orf10 in cancer development especially in urinary tumor system carcinoma. In the present study, we found that the expression of C19orf10 was significantly upregulated in BC tissues and cells. These findings implied that C19orf10 might be a cancer-promoting gene in human bladder cancer, which was consistent with the study of C19orf10 functions in liver cancer 16. The level of C19orf10 in BC tumors was significantly correlated with the pathological grade of patients with BC (*P*=0.001), indicating that C19orf10 is associated with the development of BC. In addition, we found that RNA interference-mediated C19orf10 knockdown significantly inhibited the proliferation, migration, and invasion of BC cells. Moreover, overexpression of C19orf10 significantly induced the proliferation, migration, and invasion of BC cells. Furthermore, we found that the sh-C19orf10 groups had significantly decreased xenograft volume compared to the control group. Both proliferation markers, Ki67 and PCNA, were significantly downregulated, which was consistent with our *in vitro* experimental results. These data suggest that C19orf10 is a potential oncogene in BC progression and that it has diagnostic value for patients with BC.

Accumulating studies have revealed that the PI3K/AKT/mTOR (the mammalian target of rapamycin) pathway participates in regulating cellular events, such as cell growth, adhesion, migration and survival 26-29. Our findings are consistent with the investigation on the functions of C19orf10 in liver cancer 15. Since C19orf10 can promote cell proliferation and inhibit cell apoptosis through the AKT-dependent signaling pathway in liver cancer, we speculated that C19orf10 expression might have the same effect on this pathway in BC. We found that silencing of C19orf10 significantly reduced PI3K and p-AKT expression and increased the downstream expression of p21 and p27, which regulate cell proliferation and cell cycle progression. Therefore, these results indicate that C19orf10 functions in multiple cell types through modulating the AKT signaling pathway.

Silencing C19orf10 inhibits the migration and invasion of BC cells, which is consistent with the data that C19orf10 is positively correlated with the malignant metastatic status of cancer. EMT is a key mechanism in the invasive driving process in most cases of metastatic tumor cells 24. Therefore, we tried to determine the role of C19orf10 in EMT of bladder cancer cells, and determined the expression levels of EMT-related markers and transcription factors. The Wnt/β-catenin signaling pathway has an important function in the modulation of cell pluripotency and malignant transformation, and it regulates various biological events in cancer cells, including cell growth, apoptosis, and metastasis 25. It has been reported that β-catenin/transcription factor 3 can bind to the Snail promoter and activate its transcription, thus playing a key role in β-estradiol-mediated EMT during the development of ovarian endometriosis 26. In our study, we found that after knockdown of C19orf10 in BC cells, the expression of β-catenin, Snail and Vimentin was significantly reduced, and the expression of phospho-β-catenin increased, while the protein levels of N-Cadherin and Slug did not change. Therefore, all these data indicate that β-catenin may bind to the Snail promoter to activate Vimentin expression. These findings imply that C19orf10 is involved in the regulation of BC migration and invasion, potentially through inducing EMT of BC cells via activating the Wnt/β-catenin signaling pathway. However, more experiments are needed to confirm this hypothesis.

The present study has some limitations that must be mentioned. First, there were no follow-up survival data for patients who provided the paraffin-embedded BC tissue samples for the microarray analysis. Thus, we were unable to further observe the relationship between C19orf10 expression and the survival of BC patients. Second, the occurrence and development of BC are complex processes with multiple genetic changes and multiple steps 30-32. Although this study preliminarily explored the effects of C19orf10 knockdown on the malignancy of BC cells *in vitro* and* in vivo*, there was no in-depth study on the interactions between the related molecules and signaling pathways, which will be one of our future research directions. Thirdly, we do not have data showing that human BC cells with up-regulated C19orf10 can cause more metastasis in a mouse model. This is partially due to the difficulty in establishing an orthotopic tumor metastatic model with human bladder carcinoma in mice. Since we showed that C19orf10 can promote the proliferation of human BC cell lines *in vitro* and *in vivo*, it might be difficult to distinguish that the outgrowth of BC cells with a higher C19orf10 expression level is due to enhanced potential of metastasis or due to proliferation advantage *in vivo* if a non-orthotopic approach of tumor inoculation such as intravenous (iv) injection is used. Therefore, the molecular mechanisms underlying the enhanced metastasis potential of human BC cells with higher levels of C19orf10 still need further in-depth investigations after the optimization of *in vivo* metastasis animal models.

## Conclusions

In summary, we demonstrated that the expression of C19orf10 was upregulated in BC tissues and BC cell lines and that a high level of C19orf10 expression was closely related to a high pathological grade of BC in patients. In addition, C19orf10 is a key driver of cell proliferation, migration, and invasion. Meanwhile, silencing of C19orf10 also shows an inhibitory effect on tumor growth in SW780 cell xenografts *in vivo*, suggesting that C19orf10 has a cancer-promoting effect. Moreover, the role of C19orf10 as an oncogene in BC is associated with the activation of the PI3K/AKT and Wnt/β-catenin signaling pathways. Therefore, C19orf10 is a new candidate biomarker and target for BC treatment.

## Supplementary Material

Supplementary tables.

## Figures and Tables

**Table 1:** Correlation between C19orf10 expression and clinicopathological characteristics of bladder cancer patients

Clinicopathologic variables	No. of cases	C19orf10 expression		*P* value
	(n=192)	Low (n=121)	High (n=71)	
**Gender**				0.558
Male	153	98	55	
Female	39	23	16	
**Age**				0.979
<60	89	56	33	
≥60	103	65	38	
**Grade**				<0.001
G_1-2_	145	82	63	
G_3-4_	47	39	8	
**T stage**				0.128
T_1-2_	154	93	61	
T_3-4_	38	28	10	
**Lymph node status**				0.616
N0	188	118	70	
N+	4	3	1	
