# Supplementary material for: C19orf10 promotes malignant behaviors of human bladder carcinoma cells via regulating the PI3K/AKT and Wnt/β-catenin pathways
Source: J Cancer. 2021 May 19;12(14):4341–54. doi: 10.7150/jca.56993 (PMC8176426; doi:10.7150/jca.56993)
Supplement: Supplementary file 1 — Supplementary tables. [file jcav12p4341s1.pdf]

Supplementary Table S1 The primer sequences used for qPCR in this study.

Gene name	Sequence (5′→3′)
β-actin	F: 5′-GGCACCACA CCTTCTACAATGAG-3′<br>R: 5′-GGATAGCACAGCCTGGATAGCA-3′
C19orf10	F: 5′-GGCGTCGTGCATTCCTTCT-3′<br>R: 5′-CCATTGCTCATTGGTCCCTC-3′

F, forward; R, reverse.

Supplementary Table S2 The information of the antibodies used in this study.

Antibody name	Company	Catalog #
C19orf10	Proteintech Group Inc, Wuhan, China	11353-1-AP
p21	Cell Signaling Technology, Beverly, MA, USA	2947
p27	Cell Signaling Technology, Beverly, MA, USA	3686
PI3K	Cell Signaling Technology, Beverly, MA, USA	4249
P-AKT (s473)	Cell Signaling Technology, Beverly, MA, USA	4060
AKT (pan)	Santa Cruz Biotechnology, Inc., Santa Cruz, CA, USA	4685
phospho-β-catenin (Ser33/37/Thr41)	Cell Signaling Technology, Beverly, MA, USA	9561
β-Catenin	Santa Cruz Biotechnology, Inc., Santa Cruz, CA, USA	SC-7963
E-cadherin	Cell Signaling Technology, Beverly, MA, USA	3195
N-cadherin	Cell Signaling Technology, Beverly, MA, USA	13116
Vimentin	Santa Cruz Biotechnology, Inc., Santa Cruz, CA, USA	SC-7557-R
Snail	Cell Signaling Technology, Beverly, MA, USA	3879
Slug	Cell Signaling Technology, Beverly, MA, USA	9585
β-tubulin	*Abcam,* Cambridge*, MA, USA*	ab6046
β-actin	Santa Cruz Biotechnology, Inc. Santa Cruz, CA, USA	Sc-47778

Supplementary Table S3 The target sequences of the C19orf10-specific siRNA oligos used in this study.

Number	Sequence
si#1	TGTCCAAGCTGGTGATTG
si#2	GGGAAGTCCTATCTGTACT
si#3	CCCTCTGAAAACTGAGGAA
